# Non-Invasive Brain Stimulation in Frontotemporal Dementia: A Systematic Review of Non-Pharmacological Treatment Approaches

**DOI:** 10.3390/ijms27094117

**Published:** 2026-05-04

**Authors:** Elisa Dognini, Alessia Cerino, Rosa Manenti, Maria Cotelli, Barbara Borroni

**Affiliations:** 1 Neurophysiology Unit, IRCCS Istituto Centro San Giovanni di Dio Fatebenefratelli, 25125 Brescia, Italy; edognini@fatebenefratelli.eu; 2 Department of Molecular and Translational Medicine, University of Brescia, 25125 Brescia, Italy; alessia.cerino@unibs.it; 3 Neuropsychology Unit, IRCCS Istituto Centro San Giovanni di Dio Fatebenefratelli, 25125 Brescia, Italy; rmanenti@fatebenefratelli.eu (R.M.); mcotelli@fatebenefratelli.eu (M.C.); 4 Molecular Markers Laboratory, IRCCS Istituto Centro San Giovanni di Dio Fatebenefratelli, 25125 Brescia, Italy; 5 Department of Clinical and Experimental Sciences, University of Brescia, 25125 Brescia, Italy

**Keywords:** frontotemporal dementia, FTD, NIBS, TMS, rTMS, iTBS, tES, tACS, tDCS, non-pharmacological interventions

## Abstract

Frontotemporal dementia (FTD) is a heterogeneous disorder for which disease-modifying treatments are lacking. Non-invasive brain stimulation (NIBS) has emerged as a potential therapeutic approach to modulate dysfunctional neural networks and support residual plasticity. This systematic review aims to provide an updated overview of current NIBS applications across the main clinical syndromes associated with FTD, namely behavioral variant FTD (bvFTD), semantic variant of primary progressive aphasia (svPPA), and nonfluent variant of PPA (nfvPPA). According to PRISMA guidelines, twenty-seven studies investigating NIBS interventions in major FTD phenotypes met the inclusion criteria, predominantly employing transcranial direct current stimulation (tDCS) or repetitive transcranial magnetic stimulation (rTMS). tDCS, particularly when combined with language therapy, consistently improved several language abilities in PPA, with some evidence of maintenance over time. Benefits were most consistently reported in nfvPPA, whereas effects in svPPA were more limited and domain-specific. rTMS studies showed short-term improvements in language and executive functions, especially following stimulation of left frontal regions. In bvFTD, findings were heterogeneous, with social–cognitive outcomes appearing more sensitive to stimulation, whereas global cognitive measures showed more variable effects. NIBS, particularly tDCS combined with behavioral interventions, shows symptomatic potential in selected FTD phenotypes, but methodological heterogeneity and small samples warrant larger, well-controlled trials.

## 1. Introduction

Frontotemporal dementia (FTD) is a neurodegenerative disease characterized by predominant atrophy of the frontal and/or anterior temporal cortex [1]. One of the major challenges in FTD research lies in its remarkable genetic, neuropathological, and clinical heterogeneity [2]. A positive family history, frequently consistent with an autosomal dominant pattern of inheritance, is reported in approximately 25–50% of cases, underscoring the strong genetic contribution to the disease [3]. Mutations in the *Chromosome 9 Open Reading Frame 72* (*C9orf72*), *granulin* (*GRN*), and *Microtubule-Associated Protein Tau* (*MAPT*) genes represent the most common causes of familial forms [4,5,6,7]. From a molecular standpoint, FTD is classified according to the abnormal protein aggregates identified in neurons and glial cells within affected regions [8]. Approximately 95% of both sporadic and familial cases are associated with tau (FTLD-tau) or TDP-43 (FTLD-TDP) proteinopathies [3].

Clinically, FTD manifests with progressive behavioral disturbances, cognitive decline, and language impairment. Current diagnostic criteria distinguish three main phenotypes: the behavioral variant of FTD (bvFTD) [9], the semantic variant of primary progressive aphasia (svPPA), and the non-fluent/agrammatic variant of primary progressive aphasia (nfvPPA) [10]. bvFTD is primarily characterized by executive dysfunction accompanied by profound alterations in personality, social conduct, and emotional processing, often resulting in apathy, disinhibition, loss of empathy, and socially inappropriate behaviors [9]. svPPA typically presents with progressive deficits in single-word comprehension, object knowledge, and naming [10]. In contrast, nfvPPA is mainly defined by impaired speech production, including effortful articulation and expressive agrammatism, frequently associated with difficulties in understanding syntactically complex sentences [10].

Despite its severe functional impact, FTD remains an orphan disease, as effective pharmacological interventions capable of delaying disease progression are still lacking. Therapeutic development is further complicated by the poor correspondence between clinical phenotypes and the underlying neuropathology [11]. Indeed, reliable prediction of molecular substrates is currently feasible in genetic mutation carriers, whereas in sporadic presentations the pathology cannot be established with certainty during life [2]. The absence of validated biomarkers for sporadic FTD has substantially hindered the development of disease-modifying therapies [12], and no available treatments specifically target the core pathological processes [11]. Together, these limitations emphasize the need for a deeper understanding of disease mechanisms and for alternative therapeutic strategies that may be effective across different neuropathological subtypes.

In this context, a growing body of research over the last decades has investigated non-invasive brain stimulation (NIBS) as a potential strategy to improve symptoms across FTD phenotypes by modulating dysfunctional neural networks and possibly slowing pathological processes [13]. NIBS encompasses a range of techniques characterized by different mechanisms of action and stimulation parameters. The rationale for its application is largely inspired by post-stroke rehabilitation models. Within this framework, stimulation is thought to enhance the recruitment of relatively preserved cortical regions, compensating for damaged areas or reducing suppression exerted by impaired regions [12,14]. Although FTD is a progressive disorder, early disease stages are often associated with focal dysfunction, potentially allowing residual networks to transiently support compromised functions and promote adaptive reorganization [15]. More broadly, depending on stimulation parameters, NIBS techniques can induce lasting modifications in cortical excitability that resemble long-term potentiation- or depression-like plasticity, likely mediated by glutamatergic and GABAergic intracortical mechanisms [13,16,17,18]. This capacity to leverage neuroplasticity is particularly appealing in FTD, where disease-modifying treatments remain unavailable.

Among NIBS approaches, transcranial electrical stimulation (tES), including transcranial direct current stimulation (tDCS) [19,20] and transcranial alternating current stimulation (tACS) [21], has received considerable interest in FTD. tES involves the application of low-intensity electrical currents to the scalp, inducing subthreshold shifts in neuronal membrane potentials and thereby modulating cortical excitability within targeted networks [16,20]. Through these indirect neuromodulatory effects, tES has shown potential in reducing symptom severity, particularly when combined with structured cognitive or language interventions [15].

In contrast, transcranial magnetic stimulation (TMS) delivers brief, high-intensity magnetic pulses that generate focal electric fields in the cortex, directly inducing neuronal depolarization and action potentials [13,17]. Most studies investigating NIBS in FTD have employed repetitive TMS (rTMS), in which trains of stimuli are delivered at specific frequencies over selected cortical targets [22,23,24]. 

Despite the substantial burden imposed by this condition on patients and caregivers [25], effective treatments suitable for routine clinical practice are still lacking, highlighting the need for alternative non-pharmacological approaches. Given the urgency of identifying accessible and non-invasive therapeutic options, the present systematic review aims to provide an updated overview of current evidence on NIBS interventions across the main FTD phenotypes, namely bvFTD, svPPA, and nfvPPA. Specifically, we seek to synthesize evidence on clinical and cognitive efficacy, discuss how stimulation parameters can be tailored to different therapeutic goals, and identify patient characteristics associated with treatment responsiveness. By clarifying these aspects, we wish to guide future research toward the development of more personalized neuromodulatory strategies, enabling clinically meaningful applications in this orphan disease.

## 2. Materials and Methods

### 2.1. Search Strategies and Study Selection Process

**Figure 1:**
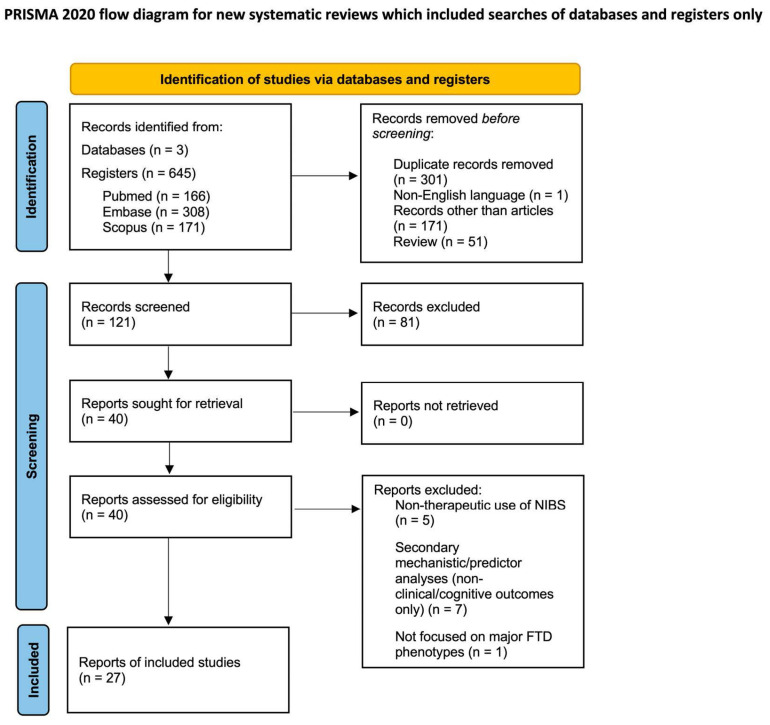
PRISMA diagram for systematic literature reviews [26]. (https://www.prisma-statement.org/prisma-2020-flow-diagram; accessed on date 23 February 2026). This work is licensed under CC BY 4.0. To view a copy of the license, visit https://creativecommons.org/licenses/by/4.0/.

This systematic review was conducted in accordance with the Preferred Reporting Items for Systematic Reviews and Meta-Analyses (PRISMA) guidelines [26]. The study selection process is summarized in the PRISMA flow diagram (Figure 1). 

The review protocol was prospectively registered in the International Prospective Register of Systematic Reviews (PROSPERO) under registration number CRD420261326465 and is publicly accessible at the PROSPERO website (https://www.crd.york.ac.uk/PROSPERO/view/CRD420261326465, accessed on 26 February 2026).

A comprehensive search was performed in the electronic databases MEDLINE (PubMed), Scopus, and Embase, with no restrictions on publication date. The search strategy used combinations of terms related to frontotemporal dementia and primary progressive aphasia, and to non-invasive brain stimulation techniques. Specifically, the following terms were searched in the Title/Abstract fields: ((frontotemporal lobar degeneration) OR (frontotemporal dementia) OR (FTD) OR (bvFTD) OR (PPA)) AND ((transcranial magnetic stimulation) OR (repetitive transcranial magnetic stimulation) OR (rTMS) OR (TMS) OR (non-invasive brain stimulation) OR (transcranial electrical stimulation) OR (TES) OR (transcranial direct current stimulation) OR (tDCS) OR (transcranial alternating current stimulation) OR (tACS) OR (transcranial random noise stimulation) OR (tRNS) OR (theta burst stimulation) OR (TBS) OR (transcranial pulse stimulation) OR (TPS) OR (transcranial ultrasound stimulation) OR (transcranial focused ultrasound stimulation) OR (tFUS) OR (low intensity focused ultrasound) OR (LiFUS) OR (tSMS) OR (ccPAS) OR (tTIS) OR (tPCS)).

Abstracts of all retrieved records were screened, and full texts of potentially eligible studies were examined. Only articles published in English were included (Figure 1). To prevent double counting, multiple records referring to the same underlying cohort/trial were collated as multiple reports of a single study.

### 2.2. Eligibility Criteria

#### 2.2.1. Inclusion Criteria

Full-length articles were included if they met the following criteria: (i) original research; (ii) studies employing non-invasive brain stimulation (NIBS) techniques, either alone or in combination with other interventions; (iii) samples including at least one major clinical phenotype of frontotemporal dementia (i.e., bvFTD, svPPA, nfvPPA); (iv) publication date prior to 4 November 2025.

#### 2.2.2. Exclusion Criteria

Studies were excluded if they met any of the following criteria: (i) written in languages other than English; (ii) conducted in animals; (iii) meta-analyses, reviews, letters, conference abstracts, case reports, or case series; (iv) focused on FTLD-spectrum disorders other than bvFTD, svPPA, or nfvPPA; (v) focused on neurodegenerative disorders not classified under FTD; (vi) focused on psychiatric conditions; (vii) lacking clinical or cognitive outcome measures; (viii) not employing multiple NIBS treatment sessions.

Case reports and descriptive case series were excluded. However, small-sample studies were included when they adopted a structured experimental design (e.g., randomized, crossover, or sham-controlled studies), and were therefore not considered descriptive case series.

### 2.3. Data Collection and Extraction

Two authors (E.D. and A.C.) independently removed duplicates and excluded review articles and conference abstracts. They then screened the remaining titles and abstracts and selected studies meeting the predefined criteria. Disagreements were resolved by consulting a third reviewer (B.B.).

Full texts of eligible articles were subsequently distributed to two reviewers (E.D. and A.C.) for detailed evaluation, leading to the final selection of studies included in this review. For each included study, data were extracted on NIBS technique and stimulation parameters, treatment features, sample characteristics, and reported clinical or cognitive outcomes.

When multiple publications clearly referred to the same cohort/trial and reported overlapping clinical/cognitive outcomes, only the most informative report was used for outcome extraction, while companion papers were consulted only to retrieve missing methodological details or numerical data; publications that did not contribute additional clinical/cognitive outcomes were not tabulated as separate entries.

Due to substantial heterogeneity in study designs, stimulation protocols, and outcome measures, studies were primarily grouped according to stimulation modality (electrical vs. magnetic). Within the electrical stimulation section, findings were further organized by clinical presentation (PPA, bvFTD, or mixed samples). A further stratification according to PPA variant was not performed, as only four studies reported results separately for the different PPA subtypes. Such stratification was not feasible for magnetic stimulation because of the limited number of available studies. Patterns of improvement, generalization, and durability of effects were qualitatively examined.

### 2.4. Quality Assessment

All studies meeting the inclusion criteria underwent a quality and risk of bias assessment using the National Heart, Lung, and Blood Institute (NHLBI) Study Quality Assessment Tools (https://www.nhlbi.nih.gov/health-topics/study-quality-assessment-tools, accessed on 2 March 2026). Each article was first classified according to its study design (e.g., randomized controlled trial, controlled intervention study, observational cohort, or cross-sectional study, before–after study with no control group, case series), and the most appropriate NHLBI tool was applied accordingly. The assessment was independently conducted by two reviewers (E.D. and A.C.), with disagreements resolved through discussion or consultation with a third reviewer (B.B.). The quality ratings assigned to each study were determined by consensus and are reported in the Supplementary Materials Figures S1 and S2.

The risk of bias assessment revealed specific methodological weaknesses across the different study designs. In before–after studies, the primary sources of bias were related to small sample sizes, the lack of blinding, and the absence of multiple post-intervention measurements. Regarding controlled intervention studies, most did not utilize an intention-to-treat analysis; furthermore, the dropout rate was often not reported or, in some cases, exceeded 20%. Notably, authors frequently failed to provide information on whether the sample size was sufficiently large to detect differences in the main outcome with at least 80% power. Finally, details concerning randomization procedures and whether treatment allocation was adequately concealed were often missing.

## 3. Results

### 3.1. Characteristics of Studies

Out of 645 records identified through database searching, 524 were removed before screening and 81 were excluded after title/abstract screening. Forty full-text reports were assessed for eligibility, and 13 were excluded with reasons. Out of 645 records identified through database searching, 524 were removed before screening and 81 were excluded after title/abstract screening. Forty full-text reports were assessed for eligibility, and 13 were excluded with reasons. The primary reasons for exclusion included a lack of specific focus on FTD phenotypes. Many studies utilized NIBS (specifically TMS) for diagnostic rather than therapeutic purposes, employing it as a neurophysiological tool to measure brain excitability and assess the influence of other variables on cortical parameters. Additionally, several studies were excluded because their primary outcomes were neurobiological or physiological (e.g., changes in the GABAergic system or functional connectivity) rather than clinical or cognitive. Finally, 27 full-text articles related to frontotemporal dementia (FTD) were included in the present systematic review. A structured qualitative synthesis was conducted. Descriptive summaries of the main findings from each study were provided to facilitate comparison, interpretation, and synthesis across studies. Moreover, the included studies [27,28,29,30,31,32,33,34,35,36,37,38,39,40,41,42,43,44,45,46,47,48,49,50,51,52] were summarized in a table providing the main details on interventions, outcome measures, and main findings (see Table 1). This approach facilitated cross-study comparison and interpretation, allowing for a comprehensive overview of the available evidence.

The twenty-seven included articles were published between 2014 and 2025, of which 22 employed tDCS and 5 employed TMS. The included articles comprised a total of 599 participants. Across primary studies, 70 participants were diagnosed with behavioral variant of FTD (bvFTD), 180 with non-fluent/agrammatic variant of primary progressive aphasia (nfvPPA), 92 with semantic variant of primary progressive aphasia (svPPA); thus, 342 participants met the FTD clinical phenotypes of interest for the present review. Some participants could not be assigned to a specific PPA or FTD phenotype [28,36,46]. One study enrolled both symptomatic and presymptomatic individuals with FTD [28]. Other samples also comprised participants with conditions beyond the FTD spectrum, such as neurodegenerative aphasia/anomia (NDA), apraxia of speech (AOS), or AD [33,44,45]. Most studies included mixed PPA cohorts, frequently encompassing the logopenic variant of primary progressive aphasia (lvPPA).

Findings specific to lvPPA were not reported separately (unless inseparable from pooled analyses), nor were findings from samples comprising NDA, AOS, or AD, in order to avoid conflating FTD and predominantly Alzheimer’s disease (AD)–related presentations. Finally, some investigations included healthy control (HC) participants, mainly to provide normative reference data for defining typical performance on neuropsychological and neurophysiological measures [42,47,49].

### 3.2. Electrical Stimulation

#### 3.2.1. Technical Parameters of Transcranial Direct Current Stimulation (tDCS) Protocols

Across studies, stimulation intensity typically ranged from 1 to 4 mA and protocols usually lasted 20–30 min/session. The most common stimulation schedule consisted of approximately 10 sessions delivered over 2 weeks, without repetition of the protocol [28,30,31,33,37,41,52]. In several investigations, this schedule was repeated two or three times (×2/×3), reflecting the application of multiple stimulation conditions or cortical targets within the same participants, rather than an extension of treatment duration [29,34,36,39,42,44,45,46,48,50,51,53]. By contrast, single-session designs were employed to probe short-term, target-specific effects, typically across different cortical regions rather than cumulative therapeutic effects [32,47,49].

#### 3.2.2. Primary Progressive Aphasia (PPA)

Across studies including individuals with PPA, anodal tDCS, most commonly combined with language therapy, has been associated with improvements in specific language and cognitive outcomes, although results varied according to stimulation site, protocol, and outcome measure [29,31,37,38,41,42,48,50,51,52]. In a single-session study in svPPA, outcomes varied markedly by target and task [49].

In randomized and crossover studies, particularly targeting the left dorsolateral prefrontal cortex (l-DLPFC) or left inferior frontal gyrus (l-IFG), tDCS paired with structured language therapy consistently augmented treatment effects. For example, in nfvPPA, anodal tDCS over the l-DLPFC enhanced individualized anomia training (oral picture naming plus reading/repetition and articulatory suppression), yielding larger and longer-lasting naming improvements compared to sham, detectable up to 12 weeks, including generalization to untrained items [30,31]. Similarly, pairing anodal l-IFG stimulation with spelling or written naming therapy in mixed PPA cohorts (including nfvPPA) improved trained-item performance under both active and sham conditions, but only active tDCS consistently supported generalization to untrained items and longer-term maintenance (up to 2 months) [50,51].

Verb-focused interventions also revealed variant- and task-specific effects. In a crossover study combining oral verb naming with spelling/written naming therapy, active tDCS produced larger immediate gains for trained verbs than sham, though this advantage was not maintained at follow-up; in contrast, robust generalization for untrained verbs persisted up to 2 months [34]. Responsiveness was also moderated by baseline language severity, with patients presenting more severe baseline impairment showing greater improvement following treatment compared to those with milder baseline deficits, although no overall main effect emerged across the cohort [39].

Exploratory analyses suggested greater responsiveness in nfvPPA than svPPA [45]. Similarly, in variant-specific analyses of IFG stimulation, active tDCS enhanced trained and untrained item performance in nfvPPA but not svPPA [51].

Finally, tDCS without concomitant language therapy also yielded measurable benefits. Benussi et al. [28] reported significant time × treatment interactions favoring active tDCS across global cognition, verbal fluency, and executive functions, alongside improvements in socio-emotional measures, including emotion recognition and behavioral inventories.

#### 3.2.3. bvFTD

Only a few studies have investigated the effects of tDCS in bvFTD, with most focusing on social-cognitive rather than language outcomes. Benussi et al. [28] reported that anodal tDCS over the left prefrontal cortex (l-PFC) improved executive function and socio-emotional measures, suggesting potential benefits for both cognitive control and emotion recognition. In contrast, Sanches et al. [47] applied single-session bilateral DLPFC tDCS without accompanying therapy and assessed language-related outcomes, global cognition, and attention/executive tasks, but observed no significant effects.

Other studies have targeted social-cognitive processes more specifically. Cotelli et al. [32] applied a single session of anodal tDCS over the medial frontal cortex to investigate theory-of-mind performance. Stimulation selectively improved accuracy for communicative intentions compared with sham, without affecting private intentions, reaction times, or global cognition, highlighting a domain-specific effect of tDCS in bvFTD.

### 3.3. Transcranial Magnetic Stimulation (TMS)

#### 3.3.1. Technical Parameters of TMS Protocols

Across studies using repetitive transcranial magnetic stimulation (rTMS) and intermittent theta-burst stimulation (iTBS), the l-DLPFC was the most frequently targeted area, followed by the l-IFG and, more rarely, right superior frontal gyrus (r-SFG) or left anterior temporal lobe (l-ATL) [27,35,38,40,43].

Stimulation was typically delivered at high frequency, ranging from 10 to 20 Hz for rTMS protocols and 50 Hz bursts for iTBS, with intensities around 90–120% of resting or active motor threshold (rMT/AMT). Most rTMS protocols consisted of 10–20 sessions distributed over 2–5 weeks [27,38,43], whereas single-session designs repeated across different cortical targets were also employed to investigate short-term, target-specific effects [40]. The iTBS protocol by Fernández-Romero et al. [35] uniquely combined an intensive 2-week daily schedule with a long-term maintenance phase of one session per week for six months, allowing evaluation of both immediate and sustained effects of stimulation over the l-DLPFC.

#### 3.3.2. Results of TMS Interventions

Across studies using TMS in FTD, stimulation was associated with changes in language, cognitive, and behavioral outcomes, although effects varied across stimulation sites, protocols, and outcome measures.

In an open-label pre–post study in an FTD cohort predominantly comprising bvFTD (with single cases of nfvPPA and svPPA), high-frequency rTMS (HF-rTMS) to the bilateral DLPFC improved global cognition, attention, executive control and behavioral symptoms [27]. However, variant-specific inferences remain limited due to the predominance of bvFTD in the cohort [27].

In PPA, rTMS produced domain- and site-specific effects. In a study using individualized rTMS targets, stimulation of active sites led to significant pre–post improvements in spontaneous speech production, object naming, reading accuracy, and repetition of syllables and disyllables, compared to control-site stimulation. Behavioral measures, including depression and apathy, also improved. Subgroup analyses indicated that nfvPPA participants benefited most from stimulation of frontal language-related regions, particularly the l-IFG, whereas svPPA participants showed larger gains following stimulation of the DLPFC or anterior temporal regions [43].

A randomized controlled trial applying HF-rTMS over the DLPFC in PPA resulted in greater improvements in naming accuracy in the active group compared to sham at 1-, 3-, and 6-month follow-up. Overall aphasia severity and functional communication were also improved in the active group. Measures of global cognition and anxiety/depression showed no significant differences between groups, and treatment response did not differ across PPA variants [38].

A crossover study comparing stimulation of the l-IFG, l-DLPFC, and vertex reported site-specific effects on language production. In nfvPPA, l-IFG stimulation increased speech productivity and improved clinical impression of change, while l-DLPFC stimulation enhanced words per minute, reduced naming latency, improved repetition performance, and also improved clinical impression. In svPPA, l-DLPFC stimulation improved reading accuracy and clinical impression of change. Narrative discourse measures revealed stimulation-dependent improvements in verbal productivity and lexical access across sites [40].

Finally, in a long-term randomized controlled trial, iTBS over the l-DLPFC followed by individualized language therapy reduced the decline in language performance compared to sham. At 3- and 6-month follow-up, participants receiving active iTBS showed less worsening on the global cognition and naming of trained items, as well as better preservation of functional independence and behavioral symptoms [35].

## 4. Discussion

Evidence summarized in this review supports the use of non-invasive brain stimulation (NIBS) in frontotemporal dementia (FTD), with reports of both short- and long-term clinical benefits. Over the past decade, NIBS has emerged as a promising symptomatic strategy across the principal FTD phenotypes. Importantly, the available data consistently point toward the need for a personalized approach. Clinically meaningful outcomes are more likely when stimulation parameters are adapted to individual patient characteristics and, when combined with cognitive therapy.

A key advantage of NIBS lies in its applicability despite the substantial neuropathological heterogeneity that characterizes FTD [11,12,13]. This feature is particularly relevant in an orphan disease in which disease-modifying therapies are lacking and clinical syndromes only partially reflect the underlying molecular substrate, especially in sporadic cases [11,12].

Both electrical and magnetic stimulation are non-invasive, generally well tolerated, and may be considered complementary techniques. Transcranial direct current stimulation (tDCS) offers practical advantages, including low cost, portability, and the possibility of home-based administration [41,54], while also allowing straightforward integration with cognitive training [37,51]. Its safety profile is favorable, with predominantly mild and transient side effects. By contrast, transcranial magnetic stimulation (TMS) provides greater spatial precision for cortical targeting. However, TMS protocols may be delivered only in hospital settings, and despite the feasibility of sham protocols, peripheral sensations may complicate blinding procedures, whereas sham conditions are often more credible with tDCS [51].

In multi-session protocols, the majority of studies demonstrate superior outcomes with active stimulation as compared to sham stimulation. The main exception comes from Borrego-Écija and colleagues [29], who observed only a trend toward clinical significance; nevertheless, they reported increased cortical activation during task-based functional Magnetic Resonance Imaging (fMRI), suggesting the presence of physiological effects despite limited clinical changes. Repeated stimulation delivered over consecutive days appears capable of inducing benefits that persist for weeks or months, supporting the clinical relevance of multi-session protocols where single-session interventions are unlikely to produce durable change [27,45].

The strongest evidence concerns the combination of neuromodulation with structured language therapy in PPA. Improvements have been described across several domains, including naming, spelling, and written production, encompassing both nouns and grammatically complex categories such as verbs [30,31,33,34,42,48,50,51,52]. While gains in trained items may occur under both active and sham conditions, active tDCS is more consistently associated with longer-lasting effects and, in some cases, generalization to untrained material [30,50]. Overall, NIBS appears to function primarily as an adjuvant that enhances the effects of cognitive/language training. Conversely, when applied as a standalone treatment, its effects on complex cognitive functions are generally modest, whereas coupling stimulation with targeted training produces more robust and selective improvements [36].

Converging neurophysiological findings further support this interpretation. Stimulation has been associated with modulation of intracortical inhibitory and facilitatory mechanisms, including changes in short-interval intracortical inhibition (SICI) and intracortical facilitation (ICF) [28,55], which reflect GABAergic and glutamatergic function and are known to be altered in FTD [17,18]. Additional studies demonstrate electrophysiological markers, such as baseline event-related potentials (ERP), may predict treatment response [33].

Indeed, several factors appear to influence efficacy. Disease severity, baseline cognitive performance, and residual structural integrity all modulate responsiveness [46,47,56]. Individuals with lower initial performance often show larger gains, possibly reflecting greater room for improvement [39]. Structural integrity of white matter [52], together with cortical volume and cortical thickness, have also emerged as important determinants [57].

Evidence from neuroimaging studies reinforces the network-based conceptualization of NIBS effects, especially with regard to tDCS. Rather than acting solely at the site of stimulation, tDCS appears to rebalance large-scale connectivity patterns disrupted by neurodegeneration, with functional changes correlating with behavioral improvement [58,59]. Greater baseline global connectivity is associated with greater disease severity, and tDCS has shown to restore more normal connectivity patterns [60]. tDCS has been shown to modulate resting-state functional connectivity between the stimulated area and interconnected nodes of the language network, with connectivity changes correlating with language gains [59].

A crucial practical issue concerns optimal targeting. Because patterns of atrophy differ across phenotypes [40,61,62], stimulation is most effective when directed toward regions that are functionally critical yet not irreversibly damaged. Lack of benefit in some svPPA cohorts likely reflects severe anterior temporal degeneration, limiting the capacity of stimulation to engage the necessary networks [45], while stimulation of language-specific regions appears more effective than stimulation of executive-support regions [49].

The necessity of our comprehensive approach is further highlighted by comparing our results with recent quantitative efforts. While several authors have reviewed NIBS in neurodegenerative diseases, most have focused on a single technique or a specific FTD variant. For instance, a recent systematic review and meta-analysis by Alhwaishel et al. [63] specifically investigated tDCS in PPA. Their findings showed that tDCS leads to a statistically significant improvement in naming objects and actions for trained items, with effects persisting in the long term; however, no significant benefits were found for untrained items, image description, or phonemic/semantic fluency compared to sham groups. They concluded that stimulation alone is insufficient, requiring combination with speech-language therapy, and noted that overall evidence is limited by small sample sizes and high heterogeneity in stimulation protocols.

In contrast to this recent work, our review adopted a broader perspective. By including not only randomized controlled trials but also pilot and exploratory studies, we aimed to provide a more exhaustive mapping of the current literature. While we also observed a higher density of studies dedicated to aphasia, our analysis identified some reports [30,50] that, unlike those in Alhwaishel’s meta-analysis, found improvements even on untrained material. Furthermore, our work extends beyond PPA to include the behavioral variant (bvFTD), which was addressed by four studies in our selection (one utilizing rTMS and three utilizing tDCS). This allows us to highlight how NIBS, and specifically repetitive TMS, can exert beneficial effects even on the behavioral and executive symptoms of FTD, a dimension that was not covered in the aforementioned meta-analysis. This broader scope justifies our qualitative synthesis as a crucial tool to bridge the gaps that quantitative analyses cannot yet cover due to the current state of the literature.

However, a critical consideration when interpreting the overall findings of this review is the methodological quality of the included literature. As detailed in our risk of bias assessment (see Section 2.4 and Supplementary Figures S1 and S2), while the majority of the evidence is derived from controlled intervention trials, a small subset of the literature consists of before–after studies without control groups. These specific designs are inherently more prone to overestimating therapeutic effects due to the lack of a sham comparator and the potential for placebo effects, which are particularly relevant in neuromodulation research.

Furthermore, even among the controlled trials, we observed frequent omissions of intention-to-treat analyses and a lack of clarity regarding randomization procedures and formal power calculations. These structural weaknesses, combined with often-unreported dropout rates, suggest that the clinical efficacy of NIBS in FTD should be viewed as promising but preliminary. The perceived benefits may be influenced by these localized methodological gaps; therefore, our conclusions must be interpreted with caution, emphasizing the urgent need for large-scale, rigorously standardized RCTs to confirm these therapeutic trends.

Overall, these findings highlight the importance of appropriate targeting and optimization of stimulation parameters. This principle provides a strong rationale for combining stimulation with cognitive tasks that actively engage the targeted networks, thereby maximizing the selectivity and efficacy of stimulation-induced effects.

Beyond conventional tDCS and rTMS, emerging techniques explored in amyotrophic lateral sclerosis (ALS) may offer important translational insights for FTD, given the recognized clinical overlap between ALS and FTD within the frontotemporal lobar degeneration (FTLD) spectrum. At present, ultrasound-based approaches such as low-intensity focused ultrasound (LiFUS) and transcranial pulse stimulation (TPS) appear more promising from a biological than a clinical standpoint, whereas transcranial static magnetic stimulation (tSMS) has yielded more concrete clinical results in ALS [64]. These observations motivate feasibility and proof-of-concept studies in FTD, ideally combining clinical endpoints with network- and biomarker-informed outcome measures. Finally, the near absence of transcranial alternating current stimulation (tACS) studies in FTD represents a significant gap. Given the well-documented alterations of oscillatory activity in dementia [36] and the ability of tACS to synchronize endogenous neural oscillations to the stimulation frequency (entrainment) [65], frequency-specific interventions may provide a more direct method to target pathophysiological mechanisms. Evidence from other neurodegenerative diseases shows both clinical and electrophysiological improvement in FTD, strongly supporting further exploration of this avenue [66,67,68]. Specifically, a recent study [66] investigated these aspects, finding that applying gamma-tACS over the precuneus in patients with Alzheimer’s Disease led to improvements in episodic memory and restored cholinergic system function. Building on these insights, a recent pilot case series by Etelämäki et al. [69] demonstrated that four days of 40 Hz tACS treatment in patients with Primary Progressive Aphasia (PPA) resulted in objective cognitive improvements, such as in verbal learning and executive functions. Notably, caregivers and family members reported persistent benefits even four weeks after the treatment, further suggesting the potential of gamma-frequency entrainment as a future therapeutic direction in FTD.

## 5. Conclusions

Overall, the included studies show promising long-term effects of non-invasive brain stimulation (NIBS) in frontotemporal dementia (FTD).

Some limitations of this review should be considered. A quantitative meta-analysis was not planned, as the objective was to provide a qualitative and clinically oriented overview of the available evidence. Consequently, conclusions rely on a narrative synthesis of findings rather than on pooled effect estimates. In addition, we excluded single-case reports and case series, which may contain informative observations but often lack adequate control conditions and generalizability. Finally, the present synthesis focused primarily on clinical and cognitive outcomes. Inclusion of neuroimaging, neurophysiological, or biological markers might have provided a more comprehensive understanding of treatment mechanisms and clarified how network-level changes relate to behavioral improvement.

Although available findings are encouraging, several methodological issues limit interpretation and comparability. Sample sizes remain small, phenotypic representation is often unbalanced, limiting inferences at the variant level, and clinical and anatomical heterogeneity are substantial. Moreover, control conditions are not always optimal, crossover designs are vulnerable to carryover effects and disease progression, follow-up durations are frequently short, and stimulation parameters vary considerably across studies. Furthermore, the limited focality of standard transcranial direct current stimulation (tDCS) montages complicates precise targeting.

Despite these limitations, the available evidence suggests that non-invasive brain stimulation, particularly tDCS and rTMS, may represent a promising symptomatic approach in frontotemporal dementia. Across studies, greater and more consistent effects have been reported when stimulation is combined with behavioral interventions, supporting a potential synergistic role of neuromodulation and cognitive training. However, the current evidence base remains limited by small sample sizes, methodological heterogeneity, and variability in outcome measures. Future research should prioritize larger, well-powered randomized controlled trials with improved phenotype stratification and greater standardization of stimulation protocols and outcome measures. Such efforts will be critical to better characterize the clinical efficacy of NIBS, refine stimulation parameters, identify patient subgroups most likely to benefit, and determine optimal targets across different stages of the disease.

## Figures and Tables

**Table 1:** Studies included in the present review.

	Technique	Protocol	Stimulation Site	Dosage	Sample	Main Findings
Electrical Stimulation						
[30]	tDCS	5 d/w, 2 w	l-DLPFC (an) Right arm (cat)	2 mA, 25 min	16 nfvPPA	Both real and sham tDCS improved naming accuracy (greater improvement in active) and functional communication (active tDCS only); maintenance over time
[50]	tDCS	3–5 d/w, 3 w × 2	l-IFG (an)	1–2 mA; 20 min	2 nfvPPA + 4 lvPPA	Both real and sham tDCS improved trained spelling; no added effect on trained items vs. untrained; improved generalization and durability (active tDCS only)
[31]	tDCS	5 d/w, 2 w	l-DLPFC (an) Right arm (cat)	2 mA, 25 min	18 nfvPPA	tDCS improved object naming (treated; untreated); no significant improvement in action naming and no maintenance at follow-up
[37]	tDCS	5 d/w, 2 w	l-IFG (an) Left occipito- parietal region (cat)	1.5 mA, 20 min	2 nfvPPA + 4 lvPPA	tDCS improved speech production, grammatical comprehension, and global performance; no effect on repetition or semantic processing
[49]	tDCS	Single session × 3	l-ATP (an), AF8 (cat); r-ATP (cat), AF7 (an); l-ATP (sham)	1.59 mA, 20 min	12 svPPA + 15 HC	Both active montages improved verbal semantic accuracy; improved performance in verbal condition with right-cathodal tDCS; no effect in visual condition or sham
[39]	tDCS	5 d/w, 2 w × 2	l-IFG (an) Left occipital region (cat)	1.5 mA, 20 min	6 nfvPPA + 1 lvPPA	No overall difference between active and sham tDCS; improved grammatical comprehension, semantic processing, and global performance with real tDCS in lower baseline performers
[44]	tDCS	10 d × 2	Left inferior parieto-temporal (an) Right fronto-orbital area (cat)	2 mA, 30 min	10 NDA/anomia	Real tDCS improved trained picture naming vs. sham; small generalization to untrained items and Digit Span (active tDCS only)
[32]	tDCS	Single session × 2	Medial frontal cortex (an) Area between Oz and Inion (cat)	1.5 mA, 10 min	16 bvFTD	Real tDCS improved accuracy for communicative intentions vs. sham; no effect on private intentions or RT
[36]	tDCS	5 d × 2	Bilateral fronto- temporal (an) Right deltoid muscle (cat)	2 mA, 20 min	8 bvFTD + 4 PPA	Real tDCS improved NPI and Simple Visual Reaction time vs. sham; no effect on language or verbal fluency
[51]	tDCS	15 d × 2	l-IFG (an) Right cheek (cat)	2 mA, 20 min	14 nfvPPA + 10 svPPA + 12 lvPPA	Real tDCS improved performance vs. sham (especially untrained items) in nfvPPA; no effect in svPPA
[34]	tDCS	10–14 d × 2	l-IFG (an) Right cheek (cat)	2 mA, 20 min	6 nfvPPA + 5 lvPPA	Real tDCS improved written verb naming vs. sham; improved generalization with longer-lasting effects; no stable maintenance for trained verbs; stronger effect for written vs. oral naming
[45]	tDCS	10 d × 3	Left inferior parieto-temporal (an); l-DLPFC (an) Right deltoid muscle (cat)	2 mA, 30 min	4 nfvPPA + 4 svPPA + 4 lvPPA	Both real montages improved trained naming vs. sham; improved durability and generalization with parieto-temporal montage; improved outcomes mainly in nfvPPA; minimal effect in svPPA
[28]	tDCS	5 d/w, 2 w	l-PFC (an) Right deltoid muscle (cat)	2 mA, 20 min	25 bvFTD + 30 PPA + 15 presymptomatic FTD	tDCS led to improvement in MMSE, phonemic fluency, TMT-A and TMT-B, Stroop, Digit symbol, Ekman, CBI; bvFTD shows improvement in TMT-A and B, and Ekman; PPA shows improvement in MMSE, phonemic fluency, TMT-A and B, Digit symbol, Ekman, and CBI; presymptomatic individuals show improvement in Stroop and Ekman
[52]	tDCS	5 d/w, 3 w	F7 (an) Right cheek (cat)	2 mA, 20 min	14 nfvPPA + 14 lvPPA + 11 svPPA	tDCS led to better improvement than sham stimulation for trained items and for untrained items up to 2 months after
[47]	tDCS	Single session × 3	l-DLPFC (an), AF8 (cat); r-DLPFC (cat), AF7 (an); l-DLPFC (sham);	1.59 mA, 20 min	12 bvFTD + 15 HC	bvFTD are worse than HC at baseline. Assessments pre-post in all three conditions do not show statistically significant differences, even between all three sessions
[29]	multifocal tDCS	5 d/w, 2 w × 2	C1, F7, FC1, FC5, Fpz, P7, PO8 (an)	2 mA, 26 min	3 lvPPA + 4 nfvPPA + 6 svPPA	Trend towards significance in trained phonemic fluency, trained semantic fluency and untrained reading speed
[53]	tDCS	12 d × 2	l-IFG (an) Right cheek (cat)	2 mA, 20 min	14 lvPPA + 13 nfvPPA + 9 svPPA	tDCS shows better performance in semantic fluency than sham stimulation, up to 2 weeks. All others tasks do not show significant differences
[41]	tDCS	10 d, 2 w	l-SMG (an) Right cheek (cat)	2 mA, 20 min	4 lvPPA + 3 nfvPPA	tDCS led to improvement in TALSA Word Span Backward and spelling
[48]	tDCS	15 d, 3 w × 2	l-IFG (an) Right shoulder (cat)	1–2 mA, 20 min	2 lvPPA + 3 nfvPPA + 3 svPPA	OANB, verbs trained: improvement in tDCS and sham at 1 week; OANB, verbs untrained: improvement in tDCS but impairment in sham (1 and 8 weeks). NAVS, sentence production: tDCS improved at 1 week; NAVS, sentence comprehension at 8 weeks BDAE: no difference between tDCS and sham, but there is a maintenance in all time points
[42]	tDCS	5 d/w, 2 w × 2	l-IFG or l-SMG (an) FP2 (cat)	1,5 mA, 20 min	3 nfvPPA + 9 lvPPA + 24 HC	tDCS-first group showed greater and more durable improvements in phonological processing and functional writing compared to sham-first group, with generalization to untrained tasks and maintenance up to 2 months; spoken language measures remained stable in both groups
[33]	HD-tDCS	5 d/w, 2 w	pre-SMA (an) + FP1, FP2, F7, F8 (cat); l-IFG (an) + T7, FP1, AF3, FC5 (cat)	1 mA, 20 min	3 nfvPPA + 3 lvPPA + 2 AOS	l-IFG stimulation led to a better phonemic fluency and production of speech pre-SMA led to a better production of speech
[46]	tDCS	5 d/w, 2 w × 3	l-DLPFC (an); bilateral DLPFC (an); sham Oz (cat)	4 mA, 20 min	17 AD + 7 FTD	Participants with a worse baseline performance improved in N-back after l-DLPFC stimulation; bilateral-DLPFC stimulation led to an improvement in MMSE at 2 weeks
Magnetic Stimulation						
[27]	rTMS	10 d	Bilateral DLPFC	10 Hz; 90% rMT/AMT	9 bvFTD + 1 nfvPPA + 1 svPPA	Improved global cognition and attention/executive measures
[43]	rTMS	15 d or 15 d + 15 d	l-IFG (9 nfvPPA), l-SFG (3 nfvPPA), l-DLPFC (1 nfvPPA + 5 sv-PPA), r-SFG (1 nfvPPA), l-anterior temporal lobe (1 svPPA), vertex	20 Hz; 100% rMT	14 nfvPPA + 6 svPPA	The active-site group improved in spontaneous speech, reading accuracy, one syllables and two syllables repetition, naming, depression, and apathy (NPI); positive impression of change
[38]	rTMS	20 d, 5 w	l-DLPFC r-DLPFC	10 Hz; 120% rMT	16 nfvPPA + 12 svPPA + 12 lvPPA	rTMS led to a greater improvement in BNT and WAB up to 6 months, and CAL up to 3 months
[35]	iTBS	10 d, 2 w + 1 d/w, 6 m	l-DLPFC	50 Hz; 120% rMT	24 nfvPPA + 12 svPPA + 27 lvPPA	MLSE at 3 and 6 months is worse in sham group; Naming (trained items) at 3 and 6 months improved in real group; IDDD improved in real group at 6 months; NPI showed less impairment in real group
[40]	rTMS	Single session × 3	l-IFG, l-DLPFC, vertex	IFG, DLPFC: 20 Hz, 100% rMT Vertex: 1 Hz, 25% rMT	14 nfvPPA + 6 svPPA	l-IFG stimulation in nfvPPA led to better performance in words per min, and clinical impression of change; l-DLPFC stimulation in nfvPPA led to better performance in words per min, naming latency, repetition, and clinical impression of change; DLPFC stimulation in svPPA improved reading accuracy, and clinical impression of change

Abbreviations: tDCS, transcranial Direct Current Stimulation; d/w, days per week; d, days; w, weeks; m, months; l, left; DLPFC, Dorsolateral Prefrontal Cortex; an, anodal; cat, cathodal; mA, milliampere; min, minutes; nfvPPA, non-fluent/agrammatic variant Primary Progressive Aphasia; MMSE, Mini-Mental State Examination; TMT-A, Trail Making Test Part A; TMT-B, Trail Making Test Part B; IFG, Inferior Frontal Gyrus; lvPPA, logopenic variant Primary Progressive Aphasia; BNT, Boston Naming Test; ATP, Anterior Temporal Pole; svPPA, semantic variant Primary Progressive Aphasia; HC, Healthy Controls; RT, Reaction Time; NDA, Neurodegenerative Anomia; bvFTD, behavioral variant Frontotemporal Dementia; NPI, Neuropsychiatric Inventory; PFC, Prefrontal Cortex; CBI, Cambridge Behavioral Inventory; SMG, Supramarginal Gyrus; TALSA, Test for Assessment of Language and Short-Term Memory in Aphasia; OANB, Object and Action Naming Battery; NAVS, Northwestern Assessment of Verbs and Sentences; BDAE, Boston Diagnostic Aphasia Examination; WAB, Western Aphasia Battery; HD-tDCS, High-density transcranial Direct Current Stimulation; pre-SMA, pre-Supplementary Motor Area; AOS, Apraxia of Speech; AD, Alzheimer’s Disease; rTMS, repetitive Transcranial Magnetic Stimulation; Hz, Hertz; rMT, resting Motor Threshold; AMT, Active Motor Threshold; SFG, Superior Frontal Gyrus; CAL, Chinese Aphasia Language test; iTBS, intermittent Theta Burst Stimulation; MLSE, Mini Linguistic State Examination; IDDD, Interview for Deterioration in Daily Living.

## Data Availability

No new data were created or analyzed in this study.

## Supplementary Materials

The following supporting information can be downloaded at: https://www.mdpi.com/article/10.3390/ijms27094117/s1.

## Abbreviations

The following abbreviations are used in this manuscript (arranged alphabetically):

AD	Alzheimer’s Disease
AMT	Active Motor Threshold
AOS	Apraxia of Speech
an	Anodal
ATP	Anterior Temporal Pole
BDAE	Boston Diagnostic Aphasia Examination
BNT	Boston Naming Test
bvFTD	behavioral variant Frontotemporal Dementia
CAL	Chinese Aphasia Language Test
cat	Cathodal
CBI	Cambridge Behavioral Inventory
d	Days
d/w	days per week
DLPFC	Dorsolateral Prefrontal Cortex
fMRI	functional Magnetic Resonance Imaging
HC	Healthy Controls
HD-tDCS	High-density transcranial Direct Current Stimulation
Hz	Hertz
IDDD	Interview for Deterioration in Daily Living
IFG	Inferior Frontal Gyrus
iTBS	intermittent Theta Burst Stimulation
l	left
lvPPA	logopenic variant Primary Progressive Aphasia
mA	milliampere
min	minutes
MLSE	Mini Linguistic State Examination
MMSE	Mini-Mental State Examination
m	months
NAVS	Northwestern Assessment of Verbs and Sentences
NDA	Neurodegenerative Anomia
nfvPPA	non-fluent/agrammatic variant Primary Progressive Aphasia
NPI	Neuropsychiatric Inventory
OANB	Object and Action Naming Battery
PFC	Prefrontal Cortex
pre-SMA	pre-Supplementary Motor Area
rMT	resting Motor Threshold
rTMS	repetitive Transcranial Magnetic Stimulation
RT	Reaction Time
SFG	Superior Frontal Gyrus
SMG	Supramarginal Gyrus
svPPA	semantic variant Primary Progressive Aphasia
TALSA	Test for Assessment of Language and Short-Term Memory in Aphasia
TMT-A	Trail Making Test Part A
TMT-B	Trail Making Test Part B
tDCS	transcranial Direct Current Stimulation
w	weeks
WAB	Western Aphasia Battery
