# Supplementary material for: Non-Invasive Brain Stimulation in Frontotemporal Dementia: A Systematic Review of Non-Pharmacological Treatment Approaches
Source: Int J Mol Sci. 2026 May 4;27(9):4117. doi: 10.3390/ijms27094117 (PMC13164393; doi:10.3390/ijms27094117)
Supplement: Supplementary file 1 [file ijms-27-04117-s001.zip › Supplementary_Materials/Figure S1.pdf]

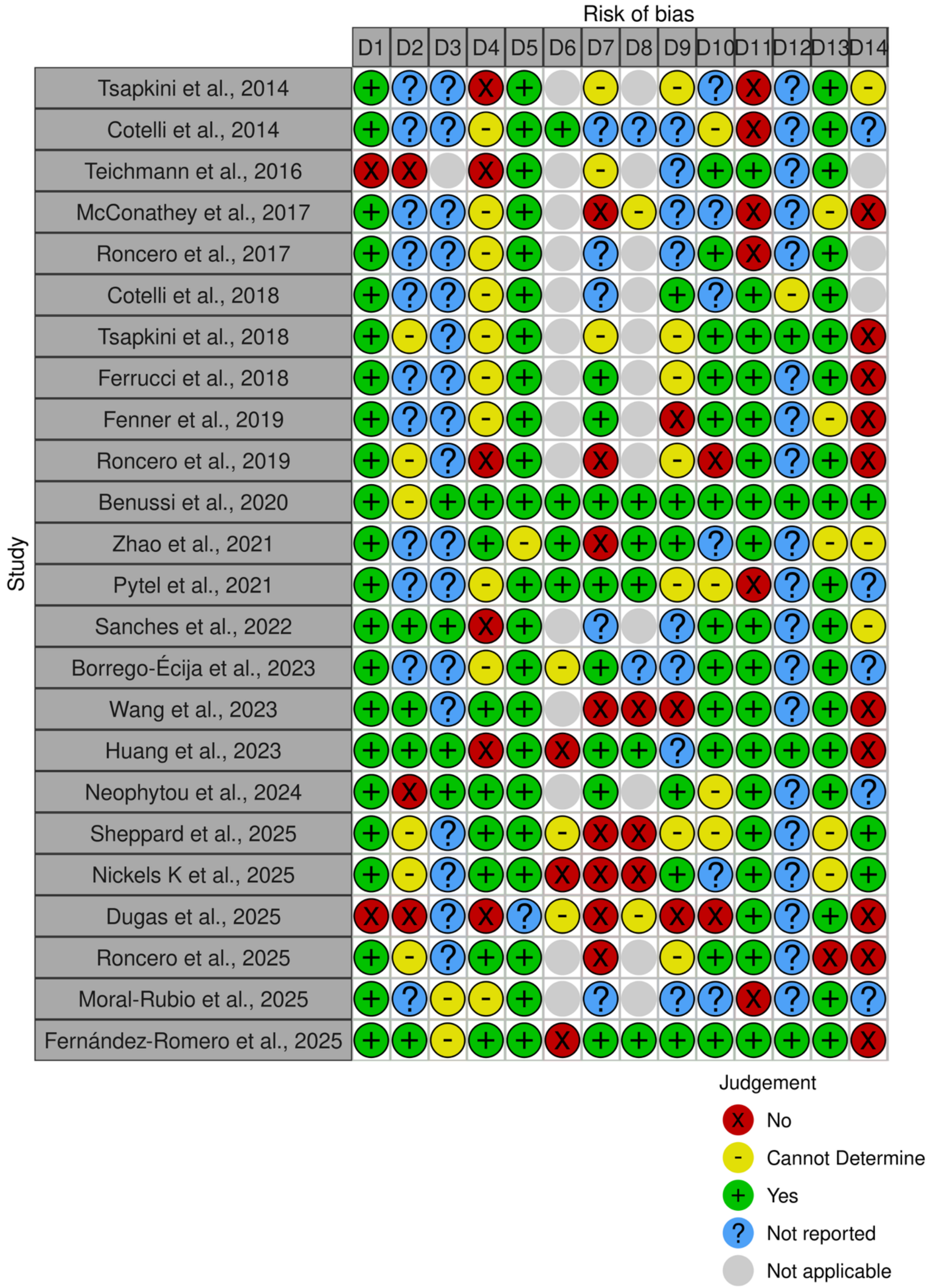

Figure S1: Risk-of-bias plot of included randomized controlled trial (adapted from [68])

References

McGuinness, L.A.; Higgins, J.P.T. Risk-of-bias VISualization (Robvis): An R Package and Shiny Web App for Visualizing Risk-of-bias Assessments. *Res. Synth. Methods* **2021**, *12*, 55–61, doi:10.1002/jrsm.1411.
