# Supplementary material for: Non-Invasive Brain Stimulation in Frontotemporal Dementia: A Systematic Review of Non-Pharmacological Treatment Approaches
Source: Int J Mol Sci. 2026 May 4;27(9):4117. doi: 10.3390/ijms27094117 (PMC13164393; doi:10.3390/ijms27094117)
Supplement: Supplementary file 1 [file ijms-27-04117-s001.zip › Supplementary_Materials/Table S1.pdf]

PRISMA 2020 for Abstracts Checklist

Section and Topic	Item #	Checklist item	Reported (Yes/No)
**TITLE**			
Title	1	Non-Invasive Brain Stimulation in Frontotemporal Dementia: A Systematic Review of Non-Pharmacological Treatment Approaches	Yes
**BACKGROUND**			
Objectives	2	This systematic review aims to provide an updated overview of current NIBS applications across the main clinical syndromes associated with FTD, namely behavioural variant of FTD (bvFTD), semantic variant of primary progressive aphasia (svPPA), and nonfluent variant of PPA (nfvPPA).	Yes
**METHODS**			
Eligibility criteria	3	Inclusion criteria: *(i)* original research; *(ii)* studies employing non-invasive brain stimulation (NIBS) techniques, either alone or in combination with other interventions; *(iii)* samples including at least one major clinical phenotype of frontotemporal dementia (i.e., bvFTD, svPPA, nfvPPA); *(iv)* publication date prior to 4 November 2025.<br>Exclusion criteria: *(i)* written in languages other than English; *(ii)* conducted in animals; *(iii)* meta-analyses, reviews, letters, conference abstracts, case reports, or case series; *(v)* focused on FTLD-spectrum disorders other than bvFTD, svPPA, or nfvPPA; *(vi)* focused on neurodegenerative disorders not classified under FTD; *(vii)* focused on psychiatric conditions; *(viii)* lacking clinical or cognitive outcome measures; *(ix)* not employing multiple NIBS treatment sessions.	Yes
Information sources	4	A comprehensive search was performed in the electronic databases MEDLINE (PubMed), Scopus, and Embase for articles published before the 04/11/2025.	Yes
Risk of bias	5	All studies meeting the inclusion criteria underwent a quality assessment using the National Heart, Lung, and Blood Institute (NHLBI) Study Quality Assessment Tools (https://www.nhlbi.nih.gov/health-topics/study-quality-assessment-tools).	Yes
Synthesis of results	6	All studies are summarized in a table (Results section).	Yes
**RESULTS**			
Included studies	7	Twenty-seven studies investigating NIBS interventions in major FTD phenotypes were included, predominantly employing transcranial direct current stimulation (tDCS) or repetitive transcranial magnetic stimulation (rTMS).	Yes
Synthesis of results	8	Evidence suggests that tDCS and rTMS are effective tools, and pairing NIBS with behavioural intervention yields greater benefits than stimulation alone. tDCS improved several language abilities especially in PPA and rTMS led to both short- and long-term improvements in language and executive functions.	Yes
**DISCUSSION**			
Limitations of evidence	9	1) Scarcity of RCT<br>2) Scarcity of FTD phenotype differentiation<br>3) Variability in stimulation targets, parameters, and cognitive tasks	Yes

PRISMA 2020 for Abstracts Checklist

Section and Topic	Item #	Checklist item	Reported (Yes/No)
Interpretation	10	NIBS are circuit-based rather than proteinopathy-specific approaches. In particular, tDCS combined with behavioural interventions show symptomatic potential in FTD. Such potential is especially relevant in an orphan disease lacking disease-modifying therapies and marked by only partial correspondence between clinical phenotypes and the underlying molecular pathology, especially in sporadic cases.	Yes
**OTHER**			
Funding	11	This research (R.M. and M.C.) is funded by the Fondazione Regionale per la Ricerca Biomedica (FRRB) – UNMET MEDICAL NEEDS — Regione Lombardia (Project title: A Multimodal Approach for Clinical Diagnosis and Treatment of Primary Progressive Aphasia, Acronym: MAINSTREAM, Project ID: 3430931) and by the Italian Ministry of Health (Ricerca Corrente).	Yes
Registration	12	The protocol for this review was registered in the International Prospective Register of Systematic Reviews (PROSPERO) under registration number CRD420261326465	Yes

Table S1: PRISMA 2020 for Abstracts Checklist [42]

From: Page MJ, McKenzie JE, Bossuyt PM, Boutron I, Hoffmann TC, Mulrow CD, et al. The PRISMA 2020 statement: an updated guideline for reporting systematic reviews. BMJ 2021;372:n71. doi: 10.1136/bmj.n71. This work is licensed under CC BY 4.0. To view a copy of this license, visit https://creativecommons.org/licenses/by/4.0/
